# Evaluating clinical importance of sensitization to Ara h 6 quantitively in Japanese children

**DOI:** 10.1016/j.waojou.2024.101001

**Published:** 2024-11-20

**Authors:** Sakura Sato, Noriyuki Yanagida, Ken-ichi Nagakura, Kyohei Takahashi, Magnus P. Borres, Motohiro Ebisawa

**Affiliations:** a Department of Allergy, Clinical Research Center for Allergy and Rheumatology, NHO Sagamihara National Hospital, Kanagawa, Japan; b Course of Allergy and Clinical Immunology, Juntendo University Graduate School of Medicine, Tokyo, Japan; c Thermo Fisher Scientific, Uppsala, Sweden; d Department of Women's and Children's Health, Uppsala University, Uppsala, Sweden

**Keywords:** Anaphylaxis, Ara h 6, Component-resolved diagnostics, Food allergy, Peanut

## Abstract

**Background:**

The clinical importance of sensitization to *Arachis hypogaea* 6 (Ara h 6) in Japanese children remains unelucidated. We aimed to quantitatively evaluate the clinical importance of sensitization to Ara h 6 in managing peanut allergy in Japanese children.

**Methods:**

We retrospectively analyzed the data of children with or without symptoms induced by an oral food challenge or home dosing of up to 3 g of peanuts. The specific immunoglobulin E (sIgE) levels against peanuts, Ara h 2, and Ara h 6 were quantified using an ImmunoCAP assay.

**Results:**

We examined 273 patients aged 4.6–9.8 years (median 6.3); 189 (69%) were male, 187 (68%) had allergies to peanuts, and 43 (16%) had anaphylactic reactions to peanuts. Ara h 6 and Ara h 2 co-sensitization was observed in 224 patients (82%). Ara h 6-sIgE levels were significantly associated with the probability of allergic reactions and anaphylaxis. The 95% probability of allergic reactions to peanuts was obtained at 44.5 kU_A_/L of Ara h 6-sIgE, but the 95% probability of anaphylaxis could not be calculated. A combination of Ara h 6 and Ara h 2 could not improve diagnostic accuracy for allergic reactions and anaphylaxis to peanuts.

**Conclusion:**

Sensitization to Ara h 6 played an important role in managing peanut allergy in Japanese children, and sIgE levels provided valuable predictive information for allergic reactions to peanuts. However, the measurement of Ara h 6 did not improve the diagnostic accuracy of anaphylaxis, and Ara h 2 alone might be sufficient for clinical evaluation in peanut allergy.

## Introduction

Peanuts (*Arachis hypogaea*) are one of the main causes of food allergies in Western countries.^1^^,^^2^ In Japan, peanuts are the fifth most frequent cause of food allergies,^3^ and accidental ingestion of peanuts is frequent in children. A correct diagnosis is essential for managing peanut allergies. However, peanut-specific immunoglobulin E (sIgE) and skin prick tests are not the most ideal for diagnosing peanut allergies.

Recent advances in molecular allergology have led to the assessment of sIgE levels in testing allergen components.^4^ Although there are 17 peanut allergens in *Arachis hypogaea* (Ara h), as stated by the World Health Organization/International Union of Immunological Societies (WAO/(IUIS) Allergen Nomenclature Sub-Committee, Ara h 2 was found to be the best predictive component for diagnosing peanut allergies.^5^, ^6^, ^7^, ^8^, ^9^ Ara h 6 belongs to the 2S albumins, as does Ara h 2, and is a major peanut allergen.^10^ In a study in Europe and the United States, Ara h 6 sensitization was associated with allergic reactions to peanuts similar to Ara h 2.^11^, ^12^, ^13^ Ara h 6 and Ara h 2 have similar molecular mass (Ara h 6, 14.5 kDa; Ara h 2, 17–19 kDa) and tertiary structures.^14^^,^^15^ However, variable IgE cross-reactivity was observed between Ara h 6 and Ara h 2,^16^ and the average rate of cross-reactive IgE represented only 17.1% of both Ara h 6 and Ara h 2-sIgE. A study in Finland showed that Ara h 6-sIgE was the most specific test for predicting moderate/severe reactions during oral food challenges (OFCs) compared to Ara h 2-sIgE.^17^ A recent study in Denmark indicated that adding Ara h 6 in the diagnostic workup would improve diagnostic accuracy of peanut allergy.^18^

A previous Japanese study evaluated the clinical relevance of sensitization to Ara h 1, 2, 3, 5, 8, and 9 and was found sensitization to Ara h 1, 2, and 3 demonstrated high specificity in diagnosing peanut allergy.^19^ Another study showed that Ara h 2-sIgE was a good predictor of diagnosing peanut allergy and was useful for managing peanut allergy.^20^ However, little is known about the clinical importance of sensitization to Ara h 6 in Japanese children. Thus, we aimed to quantitatively evaluate the clinical importance of sensitization to Ara h 6 in managing peanut allergy in Japanese children.

## Methods

### Study participants

**Fig. 1:**
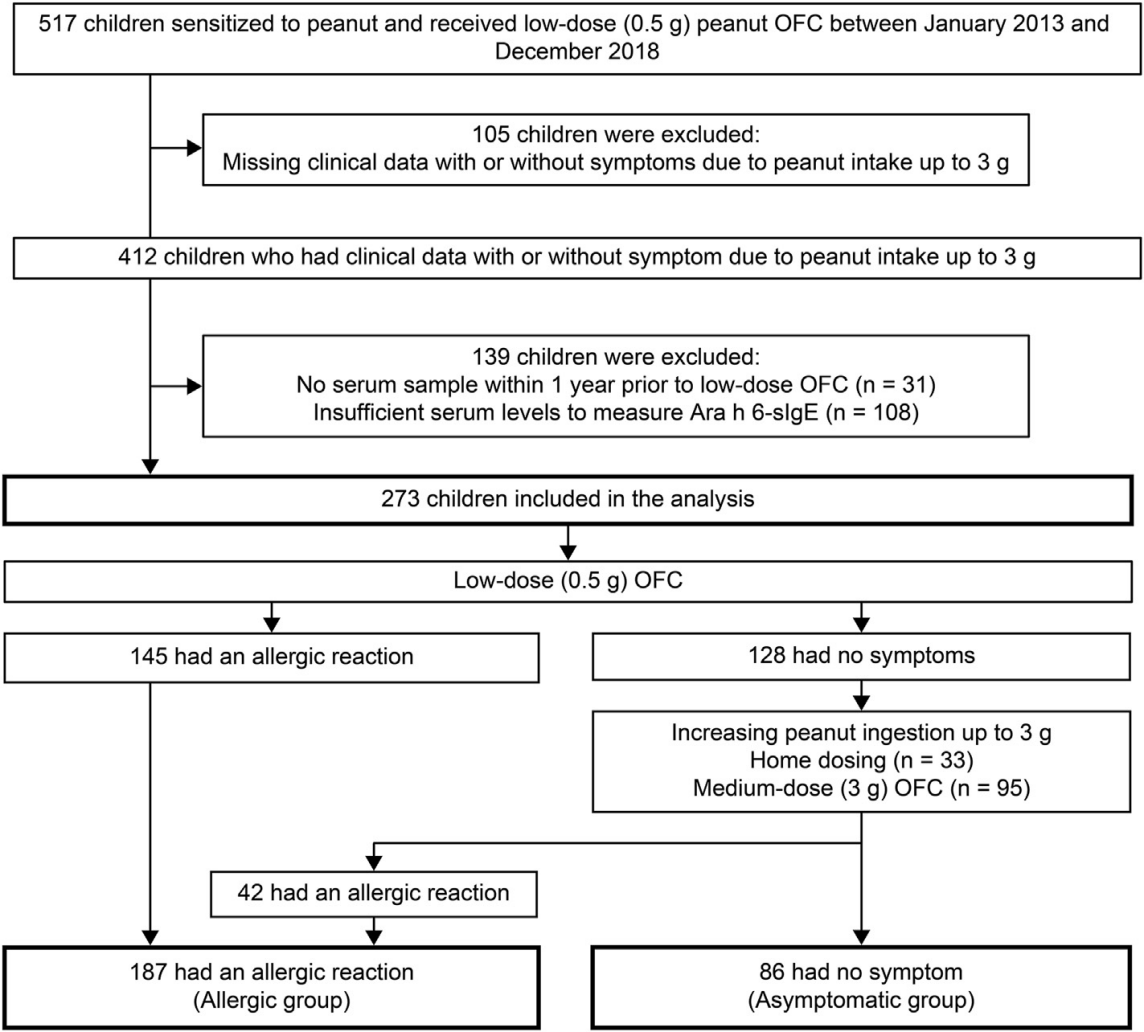
Diagnostic flow diagram of peanut allergy. Diagnosis of peanut allergy was based on the results of low-dose (0.5 g) or medium-dose (3 g) OFC or ingestion of up to 3 g of peanuts at home after passing low-dose OFC. OFC, oral food challenge

This retrospective study enrolled children sensitized to peanuts (sIgE >0.1 kU_A/_L) and those who received low-dose (0.5 g) peanut oral food challenge (OFC), at National Hospital Organization Sagamihara National Hospital between January 2013 and December 2018 (Fig. 1). Children with missing clinical data with or without symptoms due to a peanut intake of up to 3 g were excluded, and those without serum samples within 1 year before low-dose OFC or with insufficient serum amount to measure Ara h 6-sIgE.

### Oral food challenge

Open OFCs were performed in admitted patients with well-controlled allergic comorbidities. The Japanese guidelines for food allergy 2020 recommend Stepwise OFCs for diagnosing food allergy.^3^ Stepwise OFCs begin with a low-dose OFC, and the next step is performed if the previous step is negative. In this study, the total challenge dose was set at 0.5 g (low dose) and 3 g (medium dose) of peanut flour (133 mg and 795 mg of peanut protein, respectively). Challenge foods were prepared at our hospital (Supplementary Text), and were fed in increasing doses, every 30–60 min up to a cumulative dose of 0.5 or 3 g of peanuts. If an objective allergic reaction was observed, OFC was stopped. If only slight or subjective symptoms were observed, we asked the patients to ingest the peanut products again at home to investigate the reproducibility of the symptoms. Patients exhibiting allergic symptoms during OFC were treated according to the European Academy of Allergology and Clinical Immunology (EAACI) guidelines.^21^

### Determination of peanut allergy

All study participants received low-dose peanut OFC. Medium-dose peanut OFC was administered if there was no allergic reaction during the low-dose peanut OFC. Alternatively, we instructed the children to ingest up to 3 g of peanuts at home. According to the low-dose and/or medium-dose peanut OFC results or peanut ingestion at home after the low-dose peanut OFC, the study participants were divided into 2 categories: 1) Allergic group: reacted to ≤3 g of peanuts, and 2) Asymptomatic group: did not react to 3 g of peanuts (Fig. 1). Allergic reactions caused by peanut intake were judged in accordance with the Japanese Guideline for Food allergy.^3^ The definition of anaphylaxis was based on the World Allergy Organization anaphylaxis guidelines.^22^ Non-anaphylactic reactions were defined as immediate reactions without anaphylaxis.

### Serum sIgE analysis

Serum sIgE levels against whole peanuts and recombinant allergen components (Ara h 6 and Ara h 2) were measured using the ImmunoCAP assay (Thermo Fisher Scientific, Phadia, Uppsala, Sweden). Serum was collected within 1 year before low-dose peanut OFC and frozen and stored at −80 °C before analysis.

### Statistical analyses

We used GraphPad Prism 8® (GraphPad Software, Inc., San Diego, CA, USA), IBM SPSS Statistics (version 25.0; IBM Corp., Armonk, NY, USA), and R version 4.0.3 (2020, The R Foundation, Vienna, Austria). Data are expressed as median, interquartile range (IQRs), and 95% confidence interval (CIs). For comparisons between groups, the Mann–Whitney *U* test (two-tailed) was used for continuous variables and Fisher's exact test for categorical variables. The *p* values < 0.05 were considered statistically significant. Receiver operating characteristic (ROC) analyses were performed for Ara h 6, Ara h 2, and peanut sIgE. The diagnostic performance of the variables was evaluated using the area under the curve (AUC). Optimal cut-off values were estimated as the level that minimized the distance between the corresponding point on the ROC curve and the point (1, 0). Univariate and multivariate analyses were performed using logistic regression analysis. We used a history of allergic reaction and anaphylaxis to peanuts as explanatory variables, as they are considered highly related to the OFC results. Logistic regression analyses were used to create probability curves and calculate the probability of failing OFC after the logarithmic transformation of sIgE values. The calculations are based on univariate logistic regression analysis using the same method as Sampson et al.^23^ Before statistical evaluation, all sIgE values below the assay cut-off (0.10 kU_A_/L) were considered as 0.05 kU_A_/L, and all values > 100 kU_A_/L (higher limit of quantitation) were considered as 101 kU_A_/L.

### Ethical considerations

In compliance with the Declaration of Helsinki, all procedures performed in this study and the risk of OFC-induced symptoms were explained to the patients and their guardians, who provided written informed consent. Participants who had already given written consent to have their serum samples stored for future research purposes were enrolled in this study. We employed an opt-out consent process to obtain participation consent from patients and their guardians. The study design and informed consent process were approved by the Ethics Committee of the National Hospital Organization Sagamihara National Hospital (approval no. 2018-029).

## Results

### Study participants and characteristics

Among the 517 children sensitized to peanuts (sIgE >0.1 kU_A/_L) and who received low-dose peanut OFC, 273 children were enrolled in this study (Fig. 1). We excluded 244 children owing to missing clinical data with or without symptoms due to peanut intake of up to 3 g, the lack of serum samples within 1 year before low-dose OFC, or insufficient serum levels to measure Ara h 6-sIgE. These excluded patients had a lower rate of a history of immediate reaction to peanuts and anaphylaxis to peanuts than those included in the study (Supplemental Table 1).

Among the 273 patients (median age: 6.3 years), 189 (69%) were boys, 150 (55%) had a history of immediate reactions to peanuts, and 62 (23%) had a history of anaphylactic reactions (Table 1).

**Table 1:** Demographic and clinical characteristics of subjects.

	All	Allergic group	Asymptomatic group	*p*-value
Number of subjects	273	187	86	
Sex (boy)	189 (69.2%)	126 (67.4%)	62 (72.1%)	0.48
Age (years)	6.3 (4.6–9.8)	6.7 (5.3–10.2)	5.2 (3.7–7.0)	<0.001
History of immediate reaction to peanuts	150 (54.9%)	125 (66.8%)	25 (29.1%)	<0.001
History of anaphylaxis to peanuts	62 (22.7%)	56 (29.9%)	6 (7.0%)	<0.001
Comorbidities				
Bronchial asthma, current	65 (23.8%)	46 (24.6%)	19 (22.1%)	0.76
Atopic dermatitis, current	166 (60.8%)	104 (55.6%)	62 (72.1%)	0.011
Allergic rhinitis, current	71 (26.0%)	57 (30.5%)	14 (16.3%)	0.017
Total IgE (IU/mL)	669 (324–1395)	600 (301–1308)	881 (538–1745)	0.006

Data are expressed as n (%), or median values with 25%–75% interquartile ranges provided in parentheses. History of immediate reaction to peanuts and anaphylaxis to peanuts was recorded prior to the oral food challenge. Ara h, *Arachis hypogaea*; IgE, immunoglobulin E, n.s: not significant

Overall, 187 children (68%) were diagnosed with peanut allergy (allergic group), while 86 (32%) did not have any symptoms after ingesting up to 3 g of peanuts (asymptomatic group; Fig. 1; Table 1). The most common symptoms were gastrointestinal, followed by skin and mucosal, and respiratory symptoms (Supplemental Table 2). A total of 43 (23%) patients developed anaphylaxis, and 144 had non-anaphylactic reactions to peanuts. Anaphylactic patients had a higher rate of a history of anaphylaxis to peanuts than non-anaphylactic patients (Supplemental Table 3). All the patients with anaphylactic reactions were successfully treated.

### Sensitization profile to Ara h 6 and Ara h 2

**Fig. 2:**
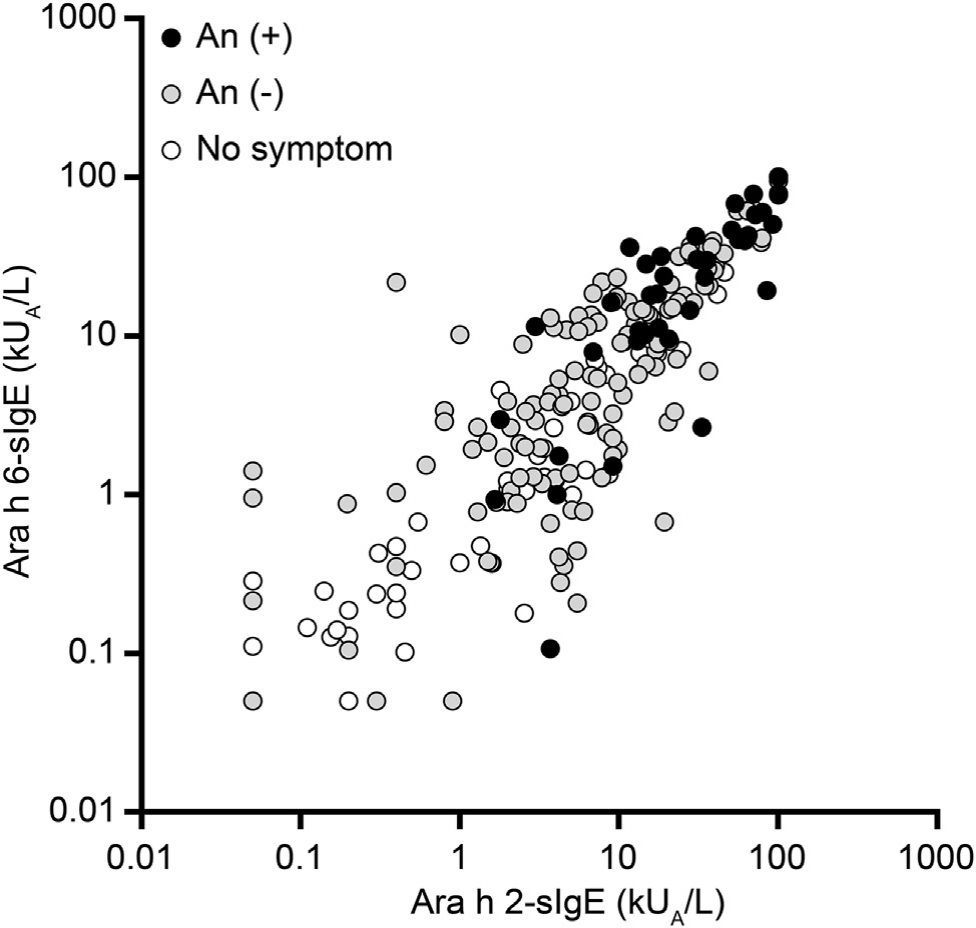
Correlation between sIgE against Ara h 6 and Ara h 2 in asymptomatic children (n = 86), those with non-anaphylactic symptoms (An (−); n = 144), and those with anaphylactic symptoms (An (+); n = 43). Asymptomatic children are indicated using filled white circles, patients with non-anaphylactic symptoms are indicated using filled gray circles, and those with anaphylactic symptoms are indicated using black circles

The sIgE levels against Ara h 6 were strongly correlated with Ara h 2-sIgE levels (r_s_ = 0.916, *p* < 0.001, Fig. 2). In our study population, sensitization (>0.1 kU_A_/L) to Ara h 6 was observed in 229 patients (84%), and that of Ara h 2 was the same number of patients (Supplementary Table 4). Ara h 6 and Ara h 2 co-sensitization was observed in 224 patients (82%). In contrast, no sensitization to either allergen was observed in 39 patients (14%). Furthermore, among the 44 patients not sensitized to Ara h 2, 5 (11%) were sensitized to Ara h 6. This discrepancy was also observed in patients who were not sensitized to Ara h 6 (n = 5, 11%).

All 43 patients who developed anaphylaxis were sensitized to both Ara h 6 and Ara h 2. Sensitization to both allergen components was more frequently observed in the allergic group than in the asymptomatic group (94% vs. 56%, *p* < 0.001). Three of the 9 allergic patients with negative Ara h 2 results were sensitized to Ara h 6. Conversely, 2 of 8 allergic patients with negative Ara h 6 were sensitized to Ara h 2.

### Relationship between sIgE levels and symptoms

The sIgE levels against Ara h 6 were higher in the allergic group than in the asymptomatic group (9.3 vs. 0.18 kU_A_/L, *p* < 0.001) (Supplemental Fig. 1). This significant difference was also observed with Ara h 2 (9.8 vs. 0.2 kU_A_/L, *p* < 0.001) and peanut sIgE (15.5 vs. 7.9 kU_A_/L, *p* < 0.001). The diagnostic accuracies of Ara h 6 and Ara h 2-sIgE were compared by calculating their respective AUCs using the ROC analysis (Table 2). Ara h 6-sIgE can be predicted for any allergic reaction similar to Ara h 2-sIgE. Optimal cut-off points for Ara h 6 and Ara h 2 were 1.5 and 3.5 kU_A_/L, respectively. The sensitivities were 78.6% and 74.9%, and the specificities were 76.7% and 74.4%. Combined with optimal cut-off points for Ara h 6 or Ara h 2, the sensitivity was 86.1% and the specificity was 72.1%.

**Table 2:** Assay performance for peanut, Ara h 2, and Ara h 6-sIgE.

	sIgE (kU_A_/L)	AUC (95% CI)	Cut-off (kU_A_/L)	Sensitivity	Specificity	PPV	NPV
Any allergic reaction	Peanut	0.63 (0.56–0.70)	>10.7 a	61.5%	61.6%	77.7%	42.4%
			>2.4	90.4%	22.1%	71.6%	51.4%
			>41.3	25.1%	90.7%	85.5%	35.8%
			>97.1	9.6%	97.7%	90.0%	33.2%
	Ara h 2	0.81 (0.75–0.87)	>3.5 a	74.9%	74.4%	86.4%	56.7%
			>0.71	90.9%	62.8%	84.2%	76.1%
			>15.3	40.0%	90.7%	90.2%	40.8%
			>48.7	15.5%	98.8%	96.7%	35.0%
	Ara h 6	0.84 (0.78–0.89)	>1.5 a	78.6%	76.7%	88.0%	62.3%
			>0.44	90.4%	64.0%	84.5%	75.3%
			>7.9	54.0%	90.7%	92.7%	47.6%
			>32.2	20.3%	97.7%	95.0%	36.1%
	Ara h 6 or Ara h 2	N.A.	>cut-off value a	86.1%	72.1%	87.0%	70.5%

Anaphylactic reaction	Peanut	0.76 (0.68–0.84)	>22.4 a	69.8%	71.3%	31.3%	92.7%
			>5.0	93.0%	30.0%	19.9%	95.8%
			>57.5	41.9%	90.0%	43.9%	89.2%
	Ara h 2	0.81 (0.75–0.87)	>13.4 a	74.4%	74.4%	35.2%	94.0%
			>3.9	90.7%	48.7%	24.8%	96.6%
			>36.4	39.5%	90.0%	42.5%	88.8%
	Ara h 6	0.81 (0.74–0.87)	>10.8 a	72.1%	73.0%	33.3%	93.3%
			>1.7	90.7%	45.2%	23.6%	96.3%
			>31.9	41.9%	90.0%	43.9%	89.2%
	Ara h 6 or Ara h 2	N.A.	>cut-off value a	81.4%	66.5%	31.3%	95.0%

Ara h, *Arachis hypogaea*; sIgE, specific immunoglobulin E; CI, confidence interval; N.A., not applicable; NPV, negative predictive value; PPV, positive predictive value.

a Optimal cut-off values were estimated as the level that minimized the distance between the corresponding point on the ROC curve and the point (1, 0)

**Fig. 3:**
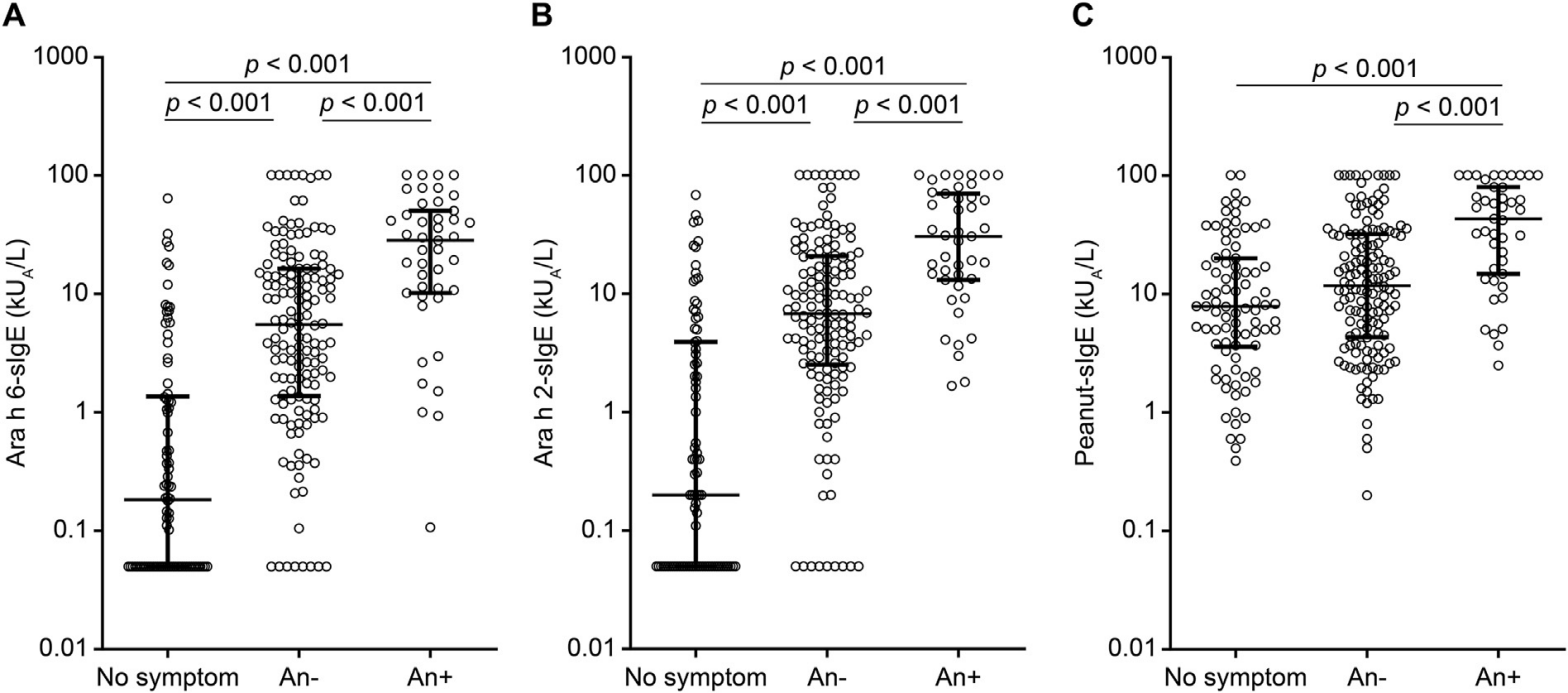
Specific IgE against (A) Ara h 6, (B) Ara h 2, and (C) peanut in asymptomatic children (n = 86), those with non-anaphylactic symptoms (An (−); n = 144), and those with anaphylactic symptoms (An (+); n = 43). Horizontal lines indicate the median and interquartile ranges. Dunn's multiple comparisons test was used to compare asymptomatic, An (−), and An (+) patients. Ara h, *Arachis hypogaea*; sIgE, specific immunoglobulin E

Ara h 6-sIgE levels were significantly higher in patients with anaphylactic symptoms than those with non-anaphylactic symptoms induced by peanuts (28.4 vs. 5.5 kU_A_/L, *p* < 0.001; Fig. 3A). Similarly, the levels of sIgE against Ara h 2 and peanuts were significantly higher in patients with anaphylactic symptoms than in those with non-anaphylactic symptoms due to peanuts (30.5 vs. 6.8 kU_A_/L, *p* < 0.001; 14.9 vs. 4.3 kU_A_/L, *p* < 0.001; Fig. 3B and C). The levels of sIgE against Ara h 6 and Ara h 2 were also significantly higher in patients with non-anaphylactic symptoms than in those without symptoms, whereas the levels of sIgE against peanuts were not significantly different between patients who had no symptoms and those with non-anaphylactic symptoms. The optimal cut-off points for Ara h 6 and Ara h 2 were 10.8 and 13.4 kU_A_/L, respectively; sensitivities were 72.1% and 74.4%, and specificities were 73.0% and 74.4%. Combined with optimal cut-off points for Ara h 6 or Ara h 2, sensitivity was 81.4% and specificity was 66.5%.

### sIgE levels for predicting allergic reactions to peanuts

**Fig. 4:**
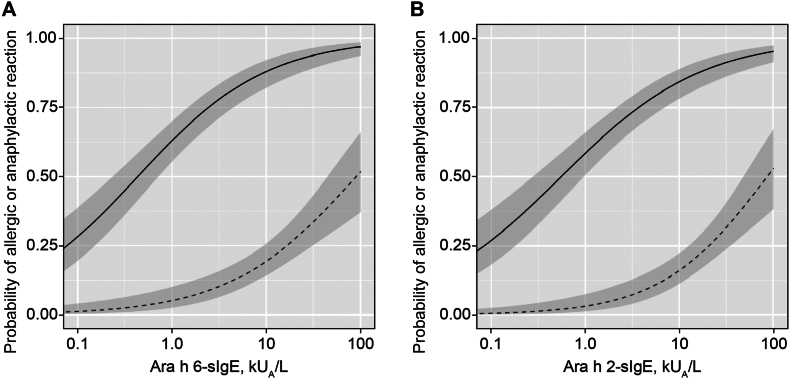
Fitted predicted probability curves for allergic and anaphylactic reactions due to peanut consumption at given (A) Ara h 6 -sIgE and (B) Ara h 2-sIgE levels. The solid line represents the probability of an allergic reaction (n = 187). The dotted line represents the probability of an anaphylactic reaction (n = 43). The surrounding-colored area indicates the 95% confidence interval (CI). Ara h, *Arachis hypogaea*; sIgE, specific immunoglobulin E

Fitted predicted probability curves for allergic reactions and anaphylaxis due to peanuts at the given Ara h 6-sIgE and Ara h 2-sIgE levels are shown in Fig. 4A and B. We calculated that a threshold Ara h 6-sIgE level of 44.5 kU_A_/L and Ara h 2-sIgE level of 88.6 kU_A_/L had a 95% probability of inducing an allergic reaction to peanuts (Table 3).

**Table 3:** Clinical efficacy of Ara h 2 and Ara h 6-sIgE in predicting allergic and anaphylactic reactions.

	Any allergic reaction		Anaphylactic reaction	
	Ara h 2-sIgE, kU_A_/L	Ara h 6-sIgE, kU_A_/L	Ara h 2-sIgE, kU_A_/L	Ara h 6-sIgE, kU_A_/L
5% probability	N.A	N.A	1.9	0.96
10% probability	N.A	N.A	4.9	3.0
50% probability	0.55	0.43	86.4	90.2
90% probability	24.5	13.7	N.A	N.A
95% probability	88.6	44.5	N.A	N.A

Ara h, *Arachis hypogaea*; sIgE, specific immunoglobulin E; N.A., not applicable

However, we could not calculate Ara h 6 and Ara h 2-sIgE levels, indicating a 95% probability of inducing anaphylaxis. A 50% predictive decision point (Ara h 6, 90.2 kU_A_/L; Ara h 2, 86.4 kU_A_/L) and a 5% predictive decision point (Ara h 6, 0.96 kU_A_/L; Ara h 2, 1.9 kU_A_/L) were observed (Table 3).

## Discussion

This study quantitatively evaluated the clinical importance of sensitization to Ara h 6 in managing peanut allergies in Japanese children, and demonstrated that most children with peanut allergies were sensitized to both Ara h 6 and Ara h 2, and increasing levels of both allergen-sIgE were related to anaphylaxis. It also revealed that Ara h 6-sIgE is a good predictive marker for the diagnosis of peanut allergy with a 95% predictive decision point but does not predict anaphylaxis due to peanut allergy.

A predictive decision point for peanut allergy can help physicians manage patients with suspected peanut allergies.^24^ In the present study, we determined a 95% predictive decision point for Ara h 6-sIgE measured by a singleplex ImmunoCAP for peanut allergy with a high degree of accuracy in diagnosing peanut allergy. Although several studies have shown that Ara h 6 is useful for predicting peanut allergy in children, these data were based on sIgE measurements using a multiplex ImmunoCAP ISAC assay.^11^^,^^12^ Recently, Brand et al investigated the correlation between Ara h 2 sIgE measured using a singleplex ImmunoCAP and a multiplex ISAC.^25^ They reported that the performance of Ara h 2-sIgE detected by ImmunoCAP® ISAC is comparable to that of Ara h 2-sIgE in ImmunoCAP. On the other hand, it has been noted that a singleplex IgE antibody assay may have more accurate quantitation and precision for measuring sIgE.^26^ Predictive decision points previously reported were analyzed based on sIgE measured by a singleplex IgE antibody assay.^20^^,^^27^, ^28^, ^29^ Regarding Ara h 6-sIgE for diagnosing peanut allergies, a study in the United Kingdom (UK) reported sIgE measured by a singleplex Immuno CAP assay. The UK study found that Ara h 6 had high diagnostic accuracy and the cut-off value at 0.32 kU_A_/L had 82% sensitivity and 90% specificity.^30^ A study in Australia evaluated the diagnostic accuracy of peanut allergen components-sIgE (Ara h 1, 2, 3, 6, 8, and 9) measured using ImmunoCAP.^31^ They showed that Ara h 6 could predict peanut OFC outcome, and the cut-off value at 0.35 kU_A_/L had 75% sensitivity and 89% specificity. A study in Denmark found that peanut-tolerance patients were more often sensitized to Ara h 2 than to Ara h 6, and Ara h 6 was better in predicting challenge outcomes than Ara h 2 by ROC analysis (AUC 0.95 vs. 0.90, *p* < 0.05).^18^ However, neither study examined predictive decision points. In contrast, the present study showed that the cut-off value at 1.5 kU_A_/L of Ara h 6 had 78.6% sensitivity and 76.7% specificity. Furthermore, a 95% predictive decision point was obtained at 44.5 kU_A_/L of Ara h 6 in a larger sample size than in these studies. Similar to a previous study using Ara h 2-sIgE,^20^ this result indicated that Ara h 6-sIgE measured using ImmunoCAP is a good predictor of allergic reactions to peanuts. The cut-off values and predictive decision points in this study differed from previous studies. Cut-off values vary according to the study population and geographic region.^25^^,^^32^ A systematic review described that the specificity of Ara h 2-sIgE was high in Northern Europe and Australia but low in North America and Asia.^33^ In addition, a history of allergic reactions alters the cut-off values for diagnosis of peanut allergy.^28^ Similar to Ara h 2, the differences in the study population may have affected the results of the current study.

A study in Finland indicated that Ara h 6 and Ara h 2 sensitization could distinguish patients with moderate to severe reactions during peanut OFC, and Ara h 6-sIgE of 0.8 ISAC Standardized Units (ISU) had 95% sensitivity and specificity.^17^ The present study also found that increased levels of Ara h 6-sIgEs correlated with the risk of anaphylaxis, similar to Ara h 2.^34^ However, the 95% predictive decision point for anaphylaxis when considering Ara h 6-sIgE was not calculated, and Ara h 6-sIgE could not predict anaphylaxis with a high probability.

Ara h 2 and Ara h 6 have similar molecular mass (Ara h 2, 17–19 kDa; Ara h 6, 14.5 kDa), similar tertiary structures, and a sequence identity of approximately 60%.^14^^,^^15^ Approximately 80% of the patients were sensitized to both Ara h 6 and Ara h 2 in our study, and sensitization of Ara h 2 and Ara h 6 may be affected by cross-allergenicity. We also confirmed a strong correlation between sIgEs against Ara h 2 and Ara h 6. This finding was similar to those in previous studies.^30^^,^^35^^,^^36^ However, Ara h 6 showed less biological activity than Ara h 2, and inhibition experiments indicated that Ara h 2 inhibited IgE-allergen binding more effectively than Ara h 6.^30^^,^^36^ Therefore, the UK study concluded that while the diagnostic accuracy of Ara h 6-sIgE was as good as Ara h 2, Ara h 2 was the dominant peanut allergen.^30^ Conversely, monosensitization to Ara h 6 was observed in patients sensitized to peanuts.^37^ A report from Iceland indicated that half of the patients sensitized to peanuts were not sensitized to Ara h 2. In Ara h 2-negative patients, sensitization to Ara h 1 and Ara h 6 caused a peanut allergy.^38^ However, the present study showed that only 4.8% (9/187) of allergic patients were Ara h 2 negative. Among these, 3 were sensitized to Ara h 6. A previous study showed that sensitization to Ara h 1 and Ara h 3 was observed in Japanese children.^19^ Therefore, the remaining 6 patients seem to be sensitized to allergens other than Ara h 6 and Ara h 2. In addition, sensitization to multiple peanut allergen components-sIgE had higher accuracy in diagnosing peanut allergy than Ara h 2-sIgE alone^39^ and all patients with severe reactions to low doses could be diagnosed with a peanut allergy when co-sensitization to Ara h 6 and Ara h 2.^17^ Although we also found that co-sensitization to Ara h 6 and Ara h 2 was associated with allergic reactions and anaphylaxis to peanuts, a combination with optimal cut-off value for Ara h 6 and Ara h 2 could not improve sensitivity and specificity. These results suggest that adding Ara h 6 does not improve the accuracy of peanut allergy diagnosis in our study cohort.

This study had limitations. First, double-blind, placebo-controlled challenges were not performed, although all allergic patients developed objective symptoms. Therefore, our conclusions are not affected. Second, the allergic group comprised children who reacted to ≤3 g of peanuts. The predictive decision points of this study did not apply to children who reacted to >3 g peanuts. Third, as the asymptomatic group did not undergo an OFC with more than 3g of peanuts, it is impossible to rule out the possibility that symptoms could be induced by consuming a much larger amount than 3g of peanuts. Finally, this study had the potential for selection bias owing to the single-center and retrospective study design. A multicenter study is needed to validate the results of this study.

In conclusion, sensitization to Ara h 6 played an important role in managing peanut allergy in Japanese children with only a few cases showing a divergence in sensitization between Ara h 6 and Ara h 2. Ara h 6-sIgE provided valuable predictive information for allergic reactions to peanuts. However, the measurement of Ara h 6 did not improve the diagnostic accuracy of anaphylaxis, and Ara h 2 alone may be sufficient for clinical evaluation in peanut allergy.

## Abbreviations

Ara h, *Arachis hypogaea*; AUC, area under the curve; CI, confidence interval; IgE, immunoglobulin E; IQRs, interquartile ranges; OFC, oral food challenges; ROC, receiver operating characteristic; sIgE, specific immunoglobulin E.

## Availability of data and materials

The data that support the findings of this study are available on request from the corresponding author, SS. The data are not publicly available due to restrictions e.g. their containing information that could compromise the privacy of research participants.

## Authors' contributions

SS designed the study and wrote the manuscript. NY and KN contributed to data collection. SS and KT performed the statistical analysis and interpretation of the results. MPB and ME were supervising editors. All authors read and approved the final manuscript.

## Ethics statement

The study design was approved by the Ethics Committee of the National Hospital Organization Sagamihara National Hospital (approval no. 2018–029).

## Authors' consent for publication

All authors have approved the manuscript and agree with the submission.

## Funding

This work was funded by the Practical Research Project for Allergic Diseases and Immunology of the Japan Agency for Medical Research and Development (AMED, 17ek0410019h0003). The funders played no role in the study design, data collection and analysis, decision to publish, or manuscript preparation.

## Declaration of competing interest

ME has received consulting fees from Novartis, Sanofi, ARS-Pharmaceuticals and honoraria from Viatris Pharmaceutical Co., Ltd. MB is employed by Thermo Fisher Scientific and Uppsala University. The remaining authors have no relevant conflicts of interest.

## Appendix A Supplementary data

Multimedia component 1 **Supplemental Figure S1**. Specific IgE against (A) Ara h 6, (B) Ara h 2, and (C) peanut in asymptomatic children (n = 86) and those with allergic children (n = 187). The median and interquartile ranges are indicated by horizontal lines. Mann-Whitney U tests were used to compare asymptomatic and allergic patients. sIgE, specific immunoglobulin E; Ara h, *Arachis hypogaea*. Multimedia component 1
