# Supplementary material for: Evaluating clinical importance of sensitization to Ara h 6 quantitively in Japanese children
Source: World Allergy Organ J. 2024 Nov 20;17(12):101001. doi: 10.1016/j.waojou.2024.101001 (PMC11617720; doi:10.1016/j.waojou.2024.101001)

**Supplementary Text**

**Food recipe used in the oral food challenge**

The challenge foods were prepared at our hospital. We used pumpkin cake or Hamburg steak containing 0.5 g (low-dose OFC) or 3.0 g (medium-dose OFC) of peanut flour (133 mg and 795 mg of peanut protein, respectively). The pumpkin cake was made from peanut flour, white sorghum flour (100% gluten-free), sugar, pumpkin, sodium bicarbonate (baking soda), and water and heated in a microwave. The Hamburg steak was made from peanut flour, pork meat, salt, and pepper, and heated in a frying pan.

**Supplemental Table 1.** Clinical and demographic characteristics of participants who received low-dose peanut oral food challenge.

|  | **Included patients**  **(n = 273)** | **Excluded patients**  **(n = 244)** | ***p*-value** |
| --- | --- | --- | --- |
| Age (years) | 6.3 (4.6–9.8) | 6.2 (4.2–9.3) | 0.427 |
| Sex (male) | 189 (69.2%) | 149 (61.1%) | 0.053 |
| History of immediate reaction to peanuts | 150 (54.9%) | 71 (29.1%) | <0.001 |
| History of anaphylaxis to peanuts | 62 (22.7%) | 39 (16.0%) | 0.046 |

Values are expressed as n (%) or median (interquartile range)

**Supplemental Table 2.** Cumulative reaction doses, symptoms, and treatments related to peanut reactions

|  | Allergic (n = 187) |
| --- | --- |
| Cumulative reacting dose of peanuts |  |
| 0.1–0.5 g | 145 (77.5%) |
| > 0.5–3 g | 42 (22.5%) |
| Symptoms |  |
| Gastrointestinal | 151 (80.7%) |
| Skin/mucosal | 86 (46.0%) |
| Respiratory | 82 (43.9%) |
| Neurological | 8 (4.3%) |
| Cardiovascular | 0 (0.0%) |
| Anaphylaxis | 43 (23.0%) |
| Treatments |  |
| Antihistamines | 116 (62.0%) |
| Steroids | 68 (36.4%) |
| β2 agonist-inhalation agents | 56 (29.9%) |
| Intramuscular adrenaline | 4 (2.1%) |

Data are presented as n (%). Anaphylaxis was defined according to the World Allergy Organization guidelines.

**Supplementary Table 3.** Comparison of clinical characteristics between anaphylactic and non-anaphylactic patients.

|  | **Anaphylactic patients** | **Non-anaphylactic patients** | ***p*-value** |  |
| --- | --- | --- | --- | --- |
| Number of subjects | 43 | 144 |  |  |
| Age (years) | 6.4 (5.4–9.8) | 7.1 (5.3–10.5) | 0.580 |  |
| Sex (male) | 30 (69.8%) | 96 (66.6%) | 0.853 |  |
| History of immediate reaction to peanuts | 30 (69.8%) | 95 (66.0%) | 0.714 |  |
| History of anaphylaxis to peanuts | 21 (48.8%) | 34 (23.6%) | 0.002 |  |
| Comorbidity |  |  |  |  |
| Bronchial asthma, current | 13 (30.2%) | 33 (22.9%) | 0.322 |  |
| Atopic dermatitis, current | 20 (46.5%) | 84 (58.3%) | 0.221 |  |
| Allergic rhinitis, current | 12 (27.9%) | 45 (31.3%) | 0.711 | |
| Total IgE (IU/mL) | 476 (299−1290) | 650 (303−1330) | 0.283 | |
| Peanut-sIgE (kU_A_/L) | 43.2 (14.9−80.2) | 11.8 (4.3−32.2) | < 0.001 | |
| Ara h 2-sIgE (kU_A_/L) | 30.5 (13.1−70.0) | 6.8 (2.5−20.8) | < 0.001 | |
| Ara h 6-sIgE (kU_A_/L) | 28.4 (10.2−50.5) | 5.5 (1.4−16.4) | < 0.001 | |

Data are expressed as n (%), or median values with 25% to 75% interquartile ranges provided in parentheses. A history of immediate reaction to peanuts and anaphylaxis to peanuts was recorded prior to the oral food challenge. The Mann–Whitney U and Fisher’s exact tests (2-tailed) were used to compare continuous and categorical data between groups, respectively. The *p*-value in the table represents a statistically significant difference between anaphylactic and non-anaphylactic cases. Ara h, *Arachis hypogaea*, IgE, immunoglobulin E.

**Supplementary Table 4.** Sensitization profile to Ara h 6 and Ara h 2

| Allergen component | sIgE (kU_A_/L) | All (n = 273) | Allergic (n = 187) | Asymptomatic (n = 86) | *p*-value |
| --- | --- | --- | --- | --- | --- |
| Ara h 6 | > 0.10 | 229 (84%) | 179 (96%) | 50 (58%) | < 0.001 |
|  | > 1.5^*^ | 167 (61%) | 147 (79%) | 20 (23%) | < 0.001 |
| Ara h 2 | > 0.10 | 229 (84%) | 178 (95%) | 51 (59%) | < 0.001 |
|  | > 3.5^*^ | 162 (59%) | 140 (75%) | 22 (26%) | < 0.001 |
| Ara h 6 and Ara h 2^†^ | > 0.10 | 224 (82%) | 176 (94%) | 48 (56%) | < 0.001 |
|  | Ara h 6 > 1.5, Ara h 2 > 3.5 | 144 (53%) | 126 (67%) | 18 (21%) | < 0.001 |

* Optimal cut-off values were estimated as the level that minimized the distance between the corresponding point on the ROC curve and the point (1, 0). ^†^ Both Ara h 6 or Ara h 2-sIgE levels were greater than each value.

**Supplemental Figures**


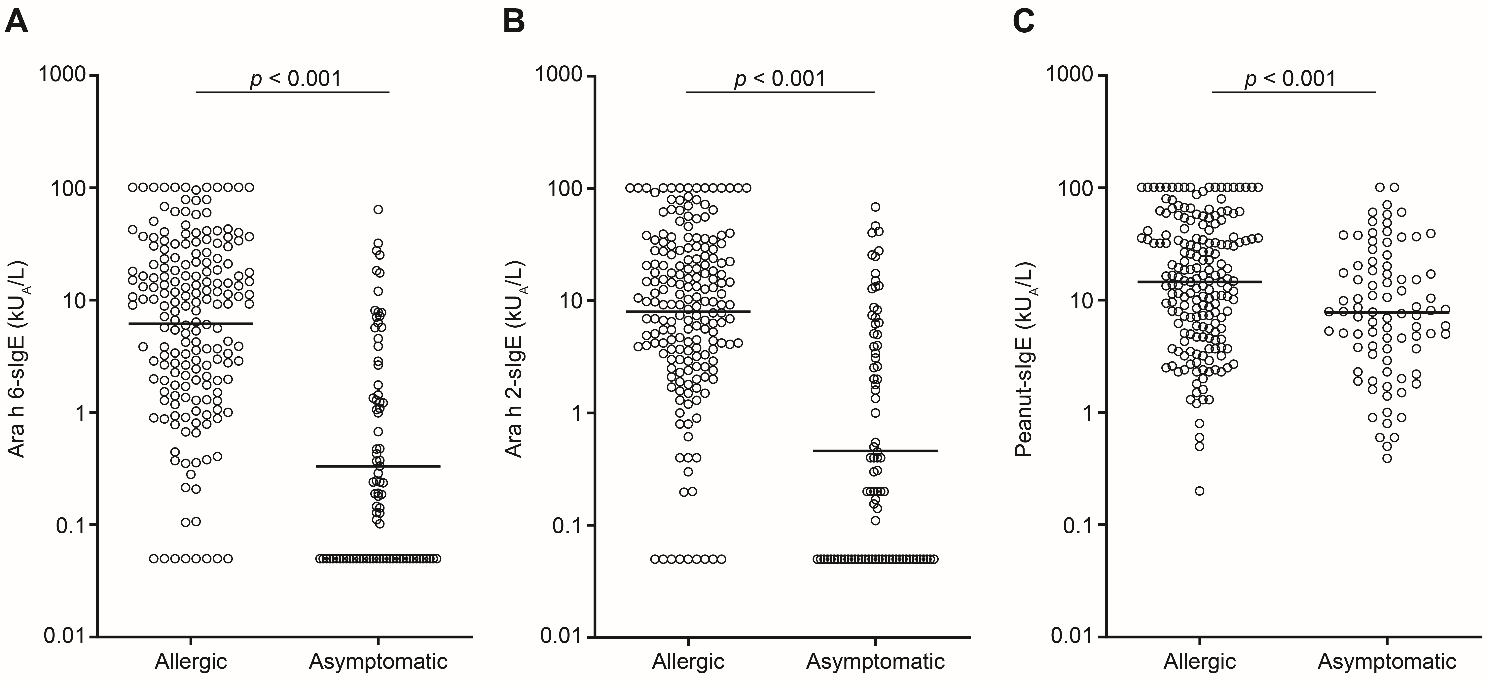

Supplement: Multimedia component 1 — Supplemental Figure S1. Specific IgE against (A) Ara h 6, (B) Ara h 2, and (C) peanut in asymptomatic children (n = 86) and those with allergic children (n = 187). The median and interquartile ranges are indicated by horizontal lines. Mann-Whitney U tests were used to compare asymptomatic and allergic patients. sIgE, specific immunoglobulin E; Ara h, Arachis hypogaea. [file mmc1.docx]
